# Smartphone imaging repository: a novel method for creating a CT image bank

**DOI:** 10.1186/s13063-022-07052-8

**Published:** 2023-01-20

**Authors:** Adrienne N. Dula, Truman J. Milling, S. Claiborne Johnston, Jayson Aydelotte, Gary W. Peil, Alec Robinson, Kaiz Asif, Stephen Pan, Sohan Parekh, Steven Warach

**Affiliations:** 1 Dell Medical School, 1601 Trinity Street, Austin, TX 78712 USA; 2 Seton Dell Medical School Stroke Institute, 1601 Trinity, 10th floor, Austin, TX 78712 USA; 3 RNDS, 1201 W. Sixth St., Suite D, Austin, TX 78705 USA; 4 Amita Health, 301 N. Madison St., Ste. 300, Joliet, IL 60435 USA

**Keywords:** Smartphone, Imaging repository

## Abstract

**Background:**

Imaging repositories are commonly attached to ongoing clinical trials, but capturing, transmitting, and storing images can be complicated and labor-intensive. Typical methods include outdated technologies such as compact discs. Electronic file transfer is becoming more common, but even this requires hours of staff time on dedicated computers in the radiology department.

**Methods:**

We describe and test an image capture method using smartphone camera video-derived images of brain computed tomography (CT) scans of traumatic intracranial hemorrhage. The deidentified videos are emailed or uploaded from the emergency department for central adjudication. We selected eight scans, mild moderate, and severe subdural and multicompartmental hematomas and mild and moderate intraparenchymal hematomas. Ten users acquired data using seven different smartphones. We measured the time in seconds it took to capture and send the files. The primary outcomes were hematoma volume measured by ABC/2, Marshall scale, midline shift measurement, image quality by a contrast-to-noise ratio (CNR), and time to capture. A radiologist and an imaging scientist applied the ABC/2 method and calculated the Marshall scale and midline shift on the data acquired on different smartphones and the PACS in a randomized order. We calculate the intraclass correlation coefficient (ICC). We measured image quality by calculating the contrast-to-noise ratio (CNR). We report summary statistics on time to capture in the smartphone group without a comparator.

**Results:**

ICC for lesion volume, midline shift, and Marshall score were 0.973 (95% CI 0.931, 0.994), 0.998 (95% CI: 0.996, 0.999), and 0.973 (0.931, 0.994), respectively. Lesion conspicuity was not different among the image types via assessment of CNR using the Friedman test, ${\lambda }^{2}$ of 24.8, *P* =  < .001, with a small Kendall’s W effect size (0.591). Mean (standard deviation) time to capture and email the video was 60.1 (24.3) s.

**Conclusions:**

Typical smartphones may produce video image quality high enough for use in a clinical trial imaging repository. Video capture and transfer takes only seconds, and hematoma volumes, Marshall scales, and image quality measured on the videos did not differ significantly from those calculated on the PACS.

## Keypoints

Question: Are smartphone video-captured computed tomography data comparable to scans on the Picture Archive and Communication System (PACS) for an imaging repository of traumatic intracranial hemorrhage?

Findings: Analyzed in multiple ways — image quality, contrast-to-noise ratio, calculation of hematoma volume, SDH thickness, midline shift, and Marshall score — smartphone-derived data were not different from PACS read scans. They can be collected and sent in about a minute.

Meaning: An image repository of traumatic intracranial hemorrhage CT scans of reasonable quality compared to PACS-read scans can be collected in 60 s.

## Introduction

Imaging repositories are valuable tools in understanding diseases but assembling them can be costly, even when attaching them to an ongoing clinical trial or drawing them from existing clinical medical records. Many clinical research sites still use compact discs to store images and mail them to trial sponsors. Electronic transfer is becoming more common [1] but is still billed at 1 to 2 h of staff time per scan. Staff often must physically go to the radiology suite to either write the CD or use other means of transmission. Smartphones have revolutionized the incorporation of imaging for consultation of specialists, making obsolete a century of lexicon in describing wounds or fractures. Clinicians routinely send deidentified images of X-rays or lacerations from their own phones or those provided by the hospital. Smartphone cameras have improved so much that entire feature-length films are shot with them [2]. Broad use of digital technology is revolutionizing health care and clinical trials, and smartphone-based imaging has been used in in vivo and ex vivo diagnostics, monitoring, and treatment (refs). Applying this pocket technology to imaging has been happening in the clinical arena for years, but it has not yet been tested as a method for assembling an image repository. The objective of this investigation is to compare smartphone video-acquired computed tomography images of traumatic intracranial hemorrhage to traditional Picture Archive and Communication System (PACS) images.

## Methods

This study is consistent with the Strengthening the Reporting of Observational Studies in Epidemiology (STROBE) criteria [3–5]. This retrospective imaging database study was not pre-registered.

### Study overview

#### Ethics

This study has regulatory and ethical approvals for waiver of consent. All data were deidentified and delinked from patient records. The shift-T function on this PACS system removes all text, which removes all identifiers. This was performed to prevent protected health information (PHI) at the source, i.e., no video contained PHI. Also, after capture and secure email, the videos were deleted from the phones; however, the video images contained no identifying data.

#### Design

Cohort study comparing PACS CT images to smartphone-captured and processed images.

#### Setting

CT scans from the trauma registry at a Level 1 trauma center in Austin, Texas.

### Image cohort selection

We selected 8 standard of care baseline computed tomography (CT) scans from traumatic intracranial hemorrhage patients in the trauma registry to represent mild, moderate, and severe subdural hematomas and multicompartmental hemorrhage, and mild and moderate isolated intraparenchymal contusions. These 8 images were captured 10 times each by smartphone video for a total of 80 smartphone videos. Additionally, one user captured data with five different smartphones (*n* = 40) for evaluation of data quality across devices. Subdural severity degree was divided by 0–5 mm width, 5–10 mm width, and > 10 mm width. Multicompartmental and intraparenchymal hemorrhage was defined as the total hematoma volume of all bleeding sites of 1–10 cc, 10–30 cc, and > 30 cc. All volumes were calculated by the simplified ABC/2 method [6, 7]. The axial CT images were acquired from the skull base to the vertex without contrast. Dose lowering techniques included adjusting the mA and/or kV per standard of care.

### Variables

The primary outcome variables were hematoma volume measured by ABC/2, Marshall scale, midline shift measurement, image quality by the contrast-to-noise ratio (CNR), and time to capture. A radiologist and an imaging scientist applied the ABC/2 method and calculated the Marshall scale and midline shift on the video images and on the PACS in a randomized order. Each dataset was assigned a number between 0 and 1 using a random number generator. The data were then presented in ascending order of random numbers. We calculate the intraclass correlation coefficient (ICC). We measured image quality by calculating the contrast-to-noise ratio (CNR). We report summary statistics on time to capture in the smartphone group without a comparator.

### Data source

Trauma registry standard of care CT scans of traumatic intracranial hemorrhage.

### Bias

We selected the scans to represent different severity of disease, which is known to affect ability to measure and is thus a better test of a new capture method, but this could be seen as a selection bias. The raters were unblinded to the study purpose which could introduce bias.

### Data collection

**Fig. 1:**
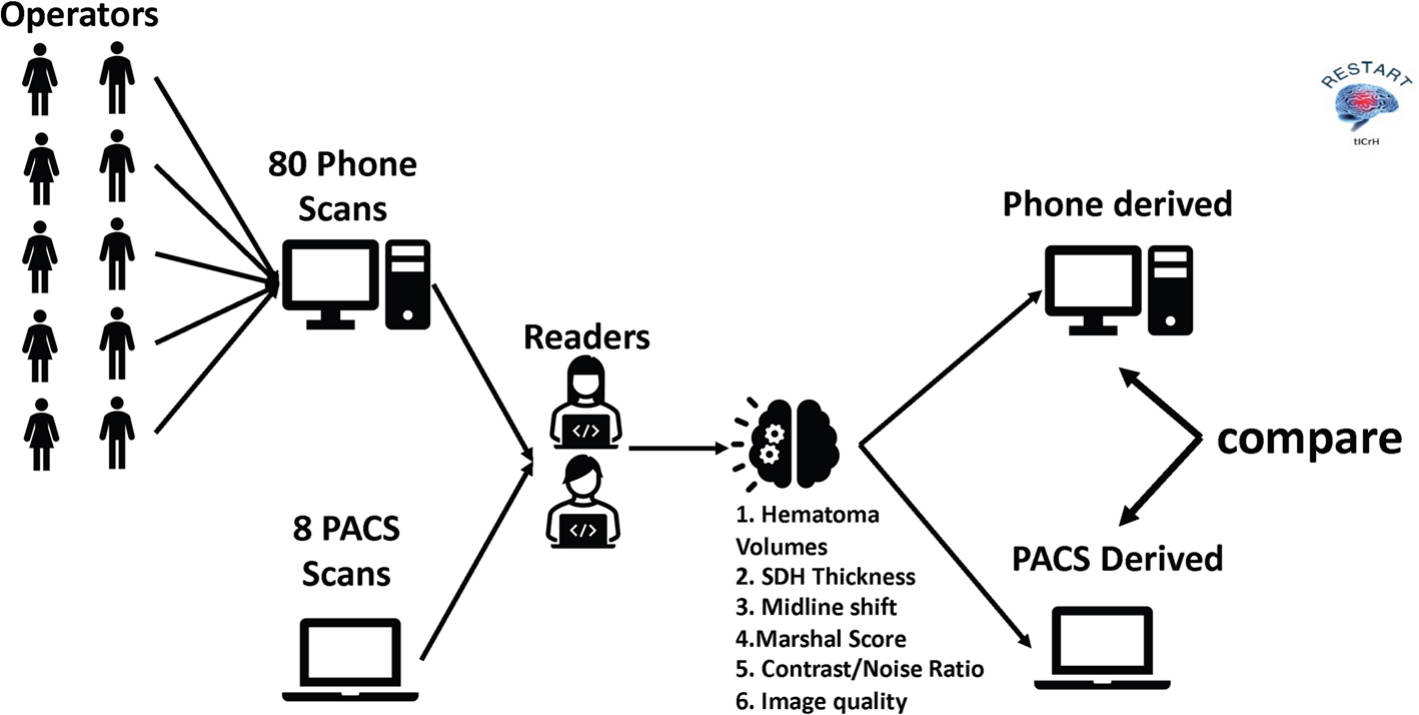
Workflow for data collection including multiple smartphone operators electronically transferring video files for post-processing and analyses. Variables collected include hematoma volume, subdural hematoma thickness, midline shift, contrast-to-noise ratio, and image quality. Nonparametric evaluation of differences across methods was performed

Data collectors included a convenience sample of emergency department (ED) staff present on a Saturday morning, including 3 attending emergency physicians, 2 emergency resident physicians, 1 emergency nurse, 1 emergency technician, 2 social workers, and a clerk. A central ED radiology reading station was used to access the Picture Archive and Communication System (PACS) via Synapse version 4.4 (Fujifilm Healthcare, Lexington, MA). The images were in DICOM format with text (and thus identifiers) hidden. Each data collector used a smartphone to record the screen while using the arrow button to scroll through axial slices of the non-contrast CT of each selected scan. The smartphones used were 3 iPhone 12 s, 2 iPhone 11 s, 1 iPhone 10, 1 iPhone 9, a Samsung Galaxy S7, a Samsung Galaxy S9, and an Android OnePlus. Data collection methods are outlined in Fig. 1.

### Image data management

Videos were saved in mp4 or mov format at native resolution and sent via a secure healthcare email platform for post processing. The resulting files were evaluated for completeness, technical quality, and adherence to the protocol requirements. Video files were converted to tagged image file (tif) format at a rate of 30 frames per second using Python version 3.7.2. Image analyses were performed using ImageJ [8] a Java-based image processing program developed at the National Institutes of Health and the Laboratory for Optical and Computational Instrumentation. All CT images were randomly presented to avoid observer recall bias.

### Image data collection

Image read data were collected and managed using REDCap (Research Electronic Data Capture) electronic data capture tools hosted at Dell Medical School at The University of Texas Austin [9, 10]. The embedded scalebar in each deidentified image was used to calibrate the scale and obtain data values that related to spatial resolution including subdural hematoma thickness, midline shift, and hematoma volume (via the ABC/2 method) [6, 7]. To quantitatively assess lesion conspicuity, image contrast to noise was calculated via

1 $$\mathrm{CNR}={}^{\left|{\mathrm{SI}}_{\mathrm{lesion}}-{\mathrm{SI}}_{\mathrm{surround}}\right|}\!\left/ \!{}_{{\sigma }_{\mathrm{surround}}}\right.$$

where SI indicates the mean signal intensity within either the lesion or surrounding tissue and $\sigma$ is the standard deviation of the image noise.

The Marshall score for diffuse head injury was recorded for each data set [11]. The ratings included (1) normal for age; (2) high/mixed density mass less than 25 cc, midline shift less than 5 mm, and basilar cisterns preserved; (3) basilar cisterns effaced and high/mixed density mass less than 25 cc, midline shift less than 5 mm, and basilar cisterns preserved; (4) midline shift greater than 5 mm; (5) evacuated mass lesion: high/mixed density mass > 25 cc which was surgically evacuated; and (6) non-evacuated mass lesion: high/mixed density mass > 25 cc not surgically treated.

### Image raters

The image raters were one imaging scientist and one radiologist working independently and reviewing the images in a randomized order, but we could not blind them as the PACS images were read on PACS and the smartphone images were read on ImageJ.

### Primary outcomes

The primary outcomes were hematoma volume measured by ABC/2, Marshall scale, midline shift measurement, image quality by the contrast-to-noise ratio (CNR), and time to capture.

### Statistics

All statistical analyses were completed in RStudio version 1.4.1106 (PBC, Boston, MA) using R version 4.1.0 (https://www.R-project.org/). Data will be made available on Zenodo along with processing script. Interrater reliability was evaluated using the intraclass correlation coefficient (ICC). Specifically, the ICC1 was calculated to reflect the amount of total variance of the measurements for each lesion type in relation to the total variance using the Pearson correlation with an $\propto$ = 0.05. To further assess the level of agreement between image analysis on the PACS vs. smartphones for quantitative measures (volume, SDH thickness, and midline shift), Bland–Altman analyses were performed with acceptable limits defined a priori to ensure the calculated critical difference was greater than the difference between each smartphone measurement and PACS.

To detect differences across the multiple phones in capturing the quantitative measures and ordinal Marshall and Likert scores for each lesion type, the non-parametric Friedman ${\lambda }^{2}$ test was used with post hoc Kendall W to determine effect size. Significance for all analyses was set at $\propto =$ 0.05.

## Results

**Fig. 2:**
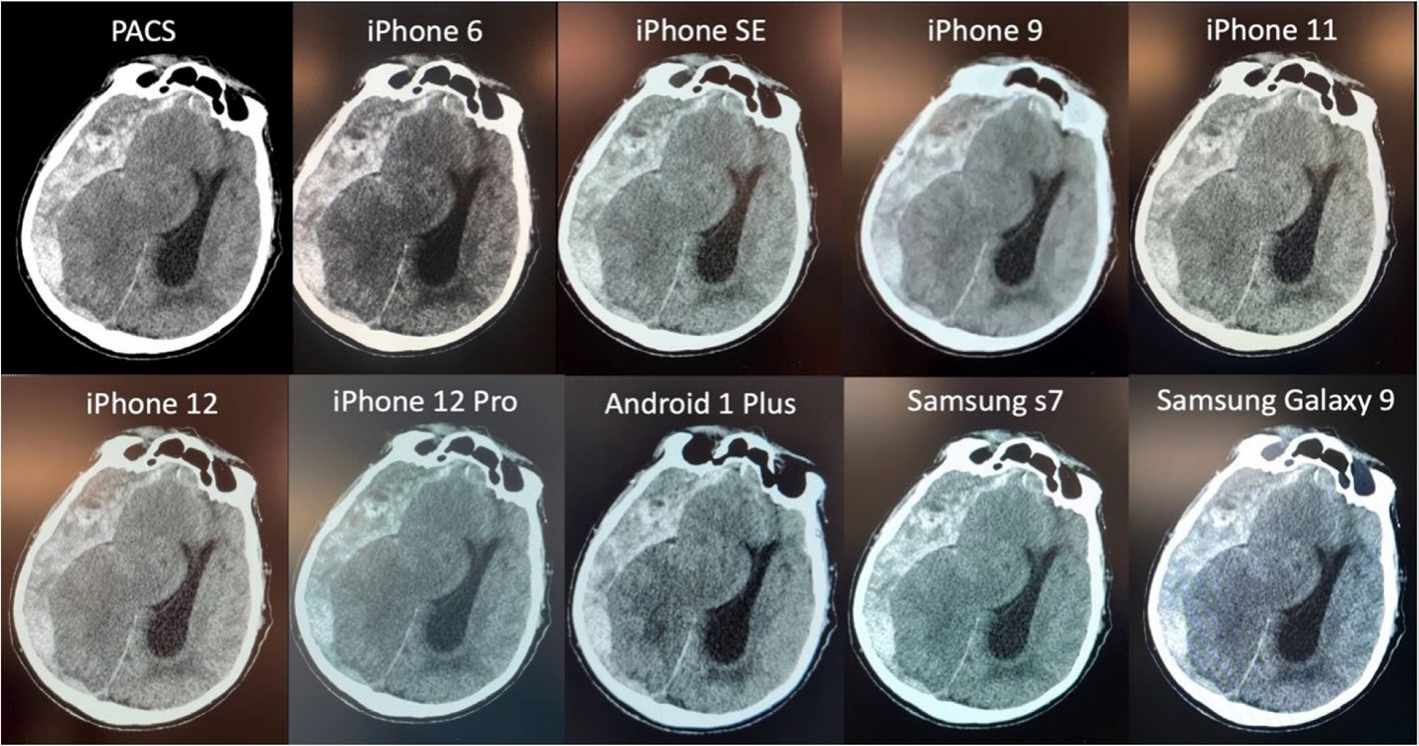
Example images of the severe multi-compartment lesion including convex subdural and intraparenchymal hematomas derived from the PACS and multiple smartphone video captures

**Fig. 3:**
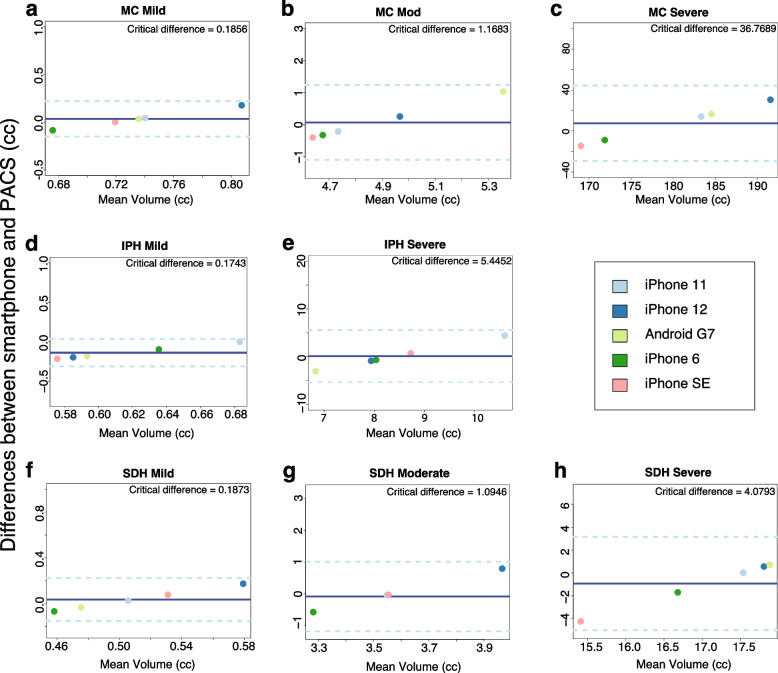
Bland–Altman plots for single user operating multiple phone models. The *y*-axis indicates the difference between hematoma volume measurement derived from PACS and various smartphone video captures on each lesion type. The difference between PACS and smartphone measures did not exceed the critical difference (gray dashed lines)

The sample of 8 index cases, 10 smartphone videos per case, was analyzed. Example results from the PACS and each smartphone model are shown in Fig. 2. Average time to capture and email the video was 60.1 s (standard deviation 24.3 s). Median (IQR) of hematoma volume, SDH thickness, midline shift, Marshall score, and CNR across smartphone models are shown in Table 1. Inter-phone reliability was high for all measures with ICC across devices for lesion volume, midline shift, and Marshall scale were 0.973 (95% CI 0.931, 0.994), 0.998 (95% CI: 0.996, 0.999), and 0.973 (0.931, 0.994), respectively (Table 2). Further analysis of volume measures (Friedman ${\lambda }^{2}$ of 41.278, *P* =  < 0.001, Kendall’s W effect size = 0.983) and Marshall score (Friedman ${\lambda }^{2}$ of 35.712, *P* =  < 0.001, Kendall’s W effect size = 0.850) ensured values derived from different smartphones are not significantly different from those measured using the PACS, (Fig. 2) and Bland–Altman plots comparing each phone type to the PACS measures (Fig. 3) support these findings.

**Table 1:** Hematoma measurements of the median (interquartile range) based on multiple smartphone recordings

Hematoma type	Volume, cc	Width, mm	Midline shift, mm	Marshall score	Contrast-to-noise
Subdural					
Mild	0.52 (0.46, 0.57)	4.0 (3.8, 4.1)	–	1 (1, 1)	5.64 (5.58, 8.34)
Moderate	3.53 (3.01, 3.54)	7.7 (7.3, 7.8)	–	2 (2, 2)	6.48 (5.61, 7.07)
Severe	17.55 (15.83, 18.09)	12.4 (12.2, 12.6)	–	2 (1, 2)	5.34 (4.86, 6.40)
Parenchymal					
Mild	0.50 (0.49, 0.59)	–	–	1 (1, 1)	3.98 (3.27, 5.32)
Severe	7.70 (7.50, 9.08)	–	–	2 (2, 2)	2.90 (2.53, 3.21)
Multi-compartment					
Mild	0.74 (0.70, 0.80)	–	–	1 (1, 1)	6.23 (5.48, 7.73)
Moderate	4.63 (4.51, 5.10)	–	–	2 (2, 2)	8.00 (7.63, 8.15)
Severe	190.41 (167.47, 192.83)	–	27.11 (26.79, 28.43)	6 (6, 6)	3.76 (3.47, 3.78)

**Table 2:** Intraclass correlation coefficient (ICC) for measures derived from the PACS and various smartphones

Attribute	ICC	ICC 95% CIs
Recording time, s	0.086	(− 0.102, 0.531)
Lesion volume, cc	0.968	(0.916, 0.993)
Midline shift, mm	0.998	(0.995, 0.999)
Marshall score	0.968	(0.915, 0.992)

The CNR was not statistically different across PACS and different phone models using the Friedman test, ${\lambda }^{2}$=24.833, *P* =  < 0.001, with an effect size of 0.591 according to Kendall’s W post hoc test.

## Discussion

Analyzed in multiple ways — image quality, contrast to noise ratio, calculation of hematoma volume, SDH thickness, midline shift, and Marshall score — smartphone scans were not inferior to PACS read scans. A 1000-patient imaging repository assembled with this method would take about 17 h of site staff time with images directly emailed to the clinical coordinating center. This is a tiny fraction of the effort using older methods, even with electronic transfers, for which coordinating centers budget 1–2 h per scan (125–250 8-h days for 1000 scans). It does not require emergency or other investigators or their research staff to physically go to the radiology department to collect and transmit images, and by replacing these methods would save time. This novel method has not previously been reported in the literature. The method has the potential to save time at the site and central coordinating center in compiling imaging repositories.

We studied deidentified, delinked scans for this analysis captured with native video cameras on several smartphone models, but obviously, the scans in a repository must be linked to patient data to be useful. Additional layers of security might be needed since the native smartphone camera video might back up to a less secure server, potentially exposing health information. We note that the scan is deidentified at the source by removing text from the PACS image, but an ideal system would have redundant security features to protect health information. We worked with a smartphone application company, RNDS, to solve this problem. RNDS was created by trauma surgeons to be a central HIPAA-compliant cloud repository for patient data along with functionalities for VOIP, video, and SMS, staff-to-staff and staff-to-patient. We adapted this technology for clinical trial purposes. It also creates the possibility of simpler methods [12] for a “meta-repository” of multiple clinical trials. We will apply these innovations to a randomized trial of timing of restarting direct oral anticoagulants after traumatic intracranial hemorrhage (Restart TICrH NCT04229758) [13].

Traumatic intracranial hemorrhage is an ideal disease in which to first study this method, as intracranial blood on CT is fairly obvious. It remains to be tested whether this approach is viable for more subtle findings such as early stroke, e.g., loss of gray-white border. Hematoma volume measurements are an important surrogate outcome [14], and clinical trial site measurements show high variability and low correlation with centrally measured scans [15]. Trials struggle collecting images for central measurement because of mislabeling, scans lost in the mail, failure to deidentify completely, etc. DICOM imaging files commonly contain metadata that is potentially identifying and difficult to remove. Our method collected more than 100 scans in less than 2 h with multiple operators and phones, only one was mislabeled, and none contained identifying information. Reading of imaging on smartphones has been previously reported [16], but there are no reports of capturing images for an imaging repository with this method.

## Limitations

We could not blind the readers to whether the images were from smartphones or PACS, as the formats differ. This might introduce bias, though probably more so against the non-traditional scans. Saving the PACS images in a video file potentially would degrade image quality, making it no longer the gold standard comparator. We are exploring converting the video into stills and loading them into the PACS for a blinded comparison. We could not test every smartphone that might be used during the trial. We tested eight different traumatic intracranial hemorrhage scans, which cannot represent the full range of this disease. All Marshall score ratings by the radiologist had a perfect agreement on the Marshall score while four reads from the imaging scientist, less familiar with the scoring system, disagreed. There may be scans on the borderline of the Marshall scale that would need a better resolution to make a definitive classification. The same may be true for mixed-density hematomas assessed for hematoma volume. It is probably true that most scans do not need this degree of resolution to be classified. For those that do, traditional methods could be used to obtain the DICOM images.

## Conclusion

Smartphone video-derived CT images of traumatic intracranial hemorrhage were non-inferior to PACS read images in terms of image quality, contrast-to-noise ratio, calculation of hematoma volume, SDH thickness, midline shift, and Marshall score. This simple novel method of capturing medical images may be a reasonable substitute for more laborious traditional methods of building a clinical trial imaging repository.

## Data Availability

Data other than images are available upon request.

## References

[1] Haak D, Page CE, Reinartz S, Krüger T, Deserno TM (2015). DICOM for clinical research: PACS-integrated electronic data capture in multi-center trials. J Digit Imaging.

[2] Phelan D. https://www.forbes.com/sites/davidphelan/2019/05/25/movie-shot-on-iphone-from-oscar-winning-director-premieres-at-cannes-film-festival-filmic-pro/?sh=54594b731b4e. Accessed 1 June 2021.

[3] von Elm E, Altman DG, Egger M, Pocock SJ, Gøtzsche PC, Vandenbroucke JP, STROBE Initiative (2007). The Strengthening the Reporting of Observational Studies in Epidemiology (STROBE)statement: guidelines for reporting observational studies. Lancet.

[4] Sharma A, Harrington RA, McClellan MB, Turakhia MP, Eapen ZJ, Steinhubl S, Mault JR, Majmudar MD, Roessig L, Chandross KJ, Green EM, Patel B, Hamer A, Olgin J, Rumsfeld JS, Roe MT, Peterson ED (2018). Using digital health technology to better generate evidence and deliver evidence-based care. J Am Coll Cardiol.

[5] Hunt B, Ruiz A, Pogue B (2021). Smartphone-based imaging systems for medical applications: a critical review. J Biomed Opt.

[6] Kothari RU, Brott T, Broderick JP, Barsan WG, Sauerbeck LR, Zuccarello M, Khoury J (1996). The ABCs of measuring intracerebral hemorrhage volumes. Stroke.

[7] Won SY, Zagorcic A, Dubinski D, Quick-Weller J, Herrmann E, Seifert V, Konczalla J (2018). Excellent accuracy of ABC/2 volume formula compared to computer-assisted volumetric analysis of subdural hematomas. PLoS ONE.

[8] Schneider CA, Rasband WS, Eliceiri KW (2012). NIH Image to ImageJ: 25 years of image analysis. Nat Methods.

[9] Harris PA, Taylor R, Thielke R, Payne J, Gonzalez N, Conde JG (2009). Research electronic data capture (REDCap)–a metadata-driven methodology and workflow process for providing translational research informatics support. J Biomed Inform.

[10] Harris PA, Taylor R, Minor BL, Elliott V, Fernandez M, O’Neal L, McLeod L, Delacqua G, Delacqua F, Kirby J, Duda SN, REDCap Consortium. The REDCap consortium: Building an international community of software platform partners. J Biomed Inform. 2019;95:103208. 10.1016/j.jbi.2019.103208 PMC7254481 31078660

[11] Marshall LF, Marshall SB, Klauber MR, Van Berkum CM, Eisenberg H, Jane JA, Luerssen TG, Marmarou A, Foulkes MA (1992). The diagnosis of head injury requires a classification based on computed axial tomography. J Neurotrauma.

[12] Lebre R, Silva LB, Costa C (2020). A cloud-ready architecture for shared medical imaging repository. J Digit Imaging.

[13] Milling TJ Jr, Warach S, Johnston SC, Gajewski B, Costantini T, Price M, Wick J, Roward S, Mudaranthakam D, Dula AN, King B, Muddiman A, Lip GYH. Restart TICrH: An Adaptive Randomized Trial of Time Intervals to Restart Direct Oral Anticoagulants after Traumatic Intracranial Hemorrhage. J Neurotrauma. 2021;38(13):1791–8. 10.1089/neu.2020.7535. Epub 2021 Apr 6. PMID: 33470152; PMCID: PMC8219199. 10.1089/neu.2020.7535 PMC8219199 33470152

[14] Mayer SA, Brun NC, Begtrup K, Broderick J, Davis S, Diringer MN, Skolnick BE, Steiner T, FAST Trial Investigators (2008). Efficacy and safety of recombinant activated factor VII for acute intracerebral hemorrhage. N Engl J Med..

[15] Hussein HM, Tariq NA, Palesch YY, Qureshi AI, ATACH Investigators (2013). Reliability of hematoma volume measurement at local sites in a multicenter acute intracerebral hemorrhage clinical trial. Stroke.

[16] Sakai K, Komatsu T, Iguchi Y, Takao H, Ishibashi T, Murayama Y (2020). Reliability of smartphone for diffusion-weighted imaging-alberta stroke program early computed tomography scores in acute ischemic stroke patients: diagnostic test accuracy study. J Med Internet Res.

